# The combined expression of Semaphorin4D and PlexinB1 predicts disease recurrence in colorectal cancer

**DOI:** 10.1186/s12885-016-2577-6

**Published:** 2016-07-25

**Authors:** Tetsuro Ikeya, Kiyoshi Maeda, Hisashi Nagahara, Masatsune Shibutani, Yasuhito Iseki, Kosei Hirakawa

**Affiliations:** Department of Surgical Oncology, Osaka City University Graduate School of Medicine, Abeno-ku, Osaka Japan

**Keywords:** Colorectal cancer, Semaphorin4D, PlexinB1, Recurrence

## Abstract

**Background:**

Binding to Sema4D and PlexinB1 induce angiogenesis and invasive growth in colorectal cancer (CRC). The expression of Semaphorin4D (Sema4D) and PlexinB1 has been shown to be related to the prognosis of patients with various malignancies. However, the correlation between the expression of Sema4D and PlexinB1 and the relapse-free survival in patients with colorectal cancer remains controversial.

**Methods:**

The study population included patients who underwent surgery for colorectal cancer (*n* = 226). The expression of Sema4D and PlexinB1 were analyzed by immunohistochemistry in tissue of stage I, II, and III colon cancers.

**Results:**

The immunohistochemical staining of colorectal cancer tissue specimens revealed that 95 (42 %) and 105 (46.4 %) of the specimens were positive for Sema4D and PlexinB1. The expression of Sema4D and PlexinB1 respectively were both found to be significantly related to stage, depth of tumor invasion, lymph node metastasis, lymphatic invasion, and venous invasion, respectively. Sixty-three patients (27.9 %) expressed both Sema4D and PlexinB1. The positive expression of both Sema4D and PlexinB1 was found to be an independent risk factor for a worse survival (HR 1.079, CI 1.013–2.868; *P* = 0.044).

**Conclusion:**

The combination of Sema4D and PlexinB1 protein detected by immunohistochemistry was therefore useful for predicting disease recurrence in CRC patients.

## Background

Worldwide, colorectal cancer (CRC) is the second highest cause of deaths among females and the third highest cause of among males with malignant neoplasms. There are 1.4 million new cases of CRC worldwide each year; 700,000 of these patients will die from CRC. With the number of CRC patients increasing each year, CRC will become the world’s most important malignancy [1].

Surgical resection is performed in most patients in whom CRC is detected. However, disease recurrence occurs in 20–25 % of patients after a curative operation [2]. In spite of treatment, most patients with recurrent CRC progress to death within a relatively short period of time. In the clinical setting, it is important to prevent recurrence to prolong survival. It is therefore helpful to understand the risk factors for recurrence. Shibutani et al. reported that the combination of the preoperative level of CEA and CA19-9 was a useful biomarker for recurrence in CRC patients after a curative operation [3]. The microsatellite instability (MSI) status has been reported to be an independent prognostic predictor of time to recurrence [4].

In this study, we focused on the Semaphorin4D (Sema4D) proteins. Semaphorins are a large family of secreted, transmembrane or glycosylphosphatidylinositol-linked proteins which contain a phylogenetically conserved extracellular “sema” domain. They are classified into eight classes, of which 3 to 7 contain vertebrate semaphorins [5, 6]. Sema4D is a transmembrane protein. Through shedding by membrane type 1-matrix metalloproteinase 1 (MT1-MMP), it transforms into a soluble form, which mainly binds to PlexinB1 [7]. PlexinB1 is single-pass transmembrane receptor for Sema4D, which is mainly expressed on endothelial cells and epithelial cells [5].

Recently, the role of Sema4D, via interaction with PlexinB1, in activities such as tumor angiogenesis and invasive growth have been discussed in relation to various types of tumors [8–10]. Thus, in the present study we investigated the expression of Sema4D and PlexinB1 in CRC tissue specimens and assessed their association with various clinicopathological factors. Finally, we clarified the potential of these variables as risk factors for CRC recurrence.

## Methods

### Patients

The study population included patients who underwent surgery for colorectal cancer between 2008 and 2011 at the Department of Surgical Oncology, Osaka City University Graduate School of Medicine, Japan. Patients who received preoperative chemotherapy were excluded from the analysis. The patients consisted of 124 males and 102 females, with a median age of 66 years (range: 21–91 years). The clinicopathological classification was determined according to the TNM classification of malignant tumors, as described by the International Union Against Cancer (UICC) [11]. The tumor stages of the patients were graded as follows: stage I (*n* = 58), stage II (*n* = 57), and stage III (*n* = 111).

### The immunohistochemical analysis

All tissues were fixed in 10 % formalin immediately after surgical resection and 6-μm-thick specimens were embedded in paraffin. The immunohistochemical determination of the Sema4D and PlexinB1 levels in the colorectal cancer cells was carried out according to the manufacturer’s instructions. In brief, the slides were deparaffinized in xylene and hydrated in decreasing concentrations of ethyl alcohol. The sections were then deparaffinized and incubated with 3 % hydrogen peroxide in methanol for 15 min to block endogenous peroxidase activity. The tissues were subsequently heated for 10 min at 105 °C by autoclaving in Target Retrieval Solution (Dako, Carpinteria, CA, USA). The sections were then washed in phosphate-buffered saline (PBS) and incubated in 10 % normal rabbit serum for 10 min to reduce nonspecific antibody binding. The specimens were incubated with antibodies to Sema4D for 1 h at room-temperature and antibodies to PlexinB1 overnight at 4 °C. They were then washed twice with PBS. The primary antibodies used for the immunohistochemical detection of anti-Sema4D and anti-PlexinB1 were Rabbit polyclonal antibody to Sema4D (SIGMA-Aldrich Ltd, Poole, UK, HPA015662, 1:150) and Rabbit polyclonal antibody to PlexinB1 (SIGMA-Aldrich Ltd, Poole, UK, HPA040586, 1:100). The sections were incubated with biotinylated rabbit anti-goat immunoglobulin G for 10 min, then washed twice with PBS. The slides were then treated with peroxidase-conjugated streptavidin reagent for 5 min and washed twice with PBS. Finally, the slides were incubated with diaminobenzidine (DAB) kit (Histofine SAB-PO Kit; Nichirei, Tokyo, Japan) for 180 s for Sema4D antibodies, and 150 s for PlexinB1 antibodies, then counterstained with Mayer’s hematoxylin and mounted.

### The evaluation of immunostaining for Sema4D

**Fig. 1:**
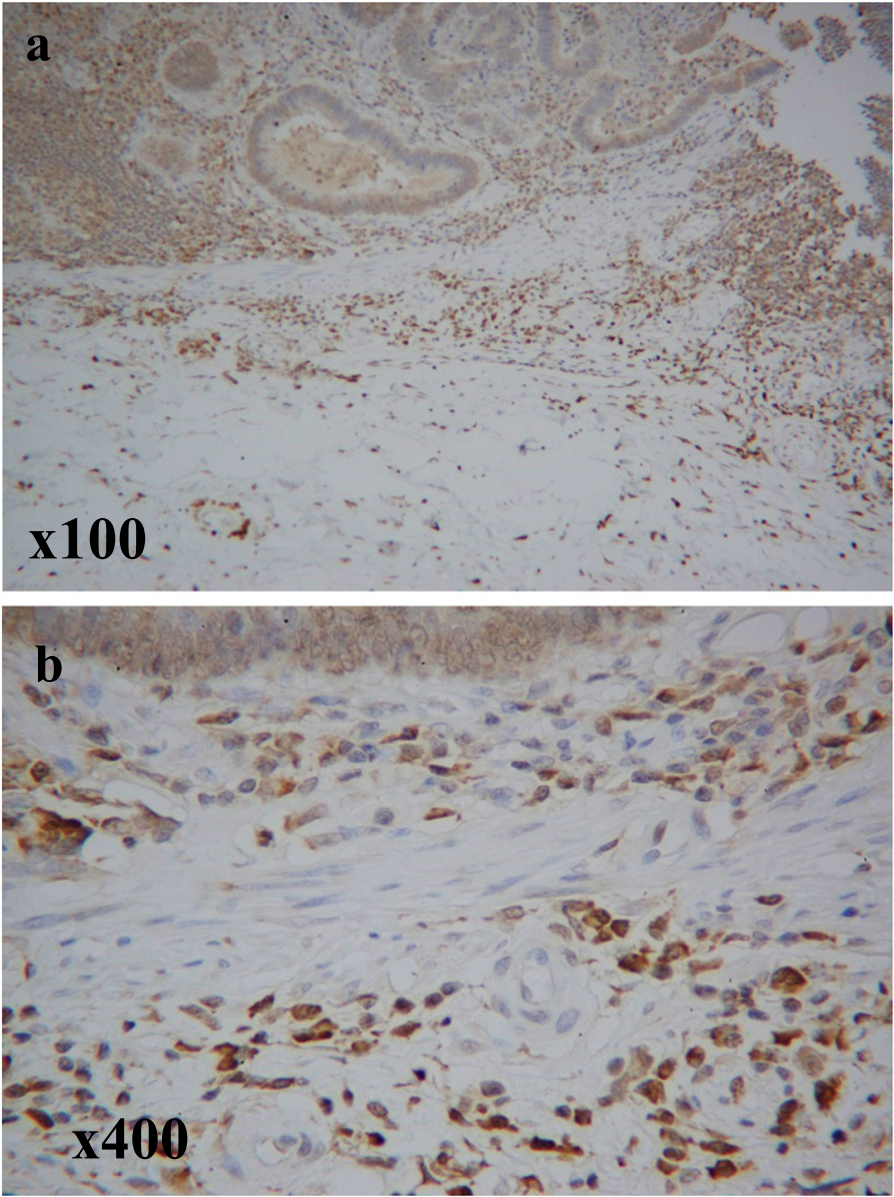
Immunohistochemical staining for Semaphorin4D. The immunohistochemical evaluation of Sema4D-positive cells in colorectal cancer specimens. The positive staining of inflammatory cells was observed in the tumor stroma. **a** The photograph was taken at a magnification of ×100, **b** The photograph was taken at a magnification of ×400

We counted the total number of infiltrating inflammatory cells in the tumor stroma in three independent high-power fields (×400) for each tissue sample. The positive Sema4D staining of inflammatory cells was observed in the tumor stroma (Fig. 1). We then calculated the percentage of Sema4D-positive cells among the total number of inflammatory cells. The specimens were then divided into three grades, according to the degree of positivity as follows: grade 1 (0–25 % positive), grade 2 (26–50 % positive) and grade 3 (51–100 % positive). For the statistical analyses, grades 1 and 2 were defined as negative, and grade 3 was defined as positive [9].

### The evaluation of immunostaining for PlexinB1

**Fig. 2:**
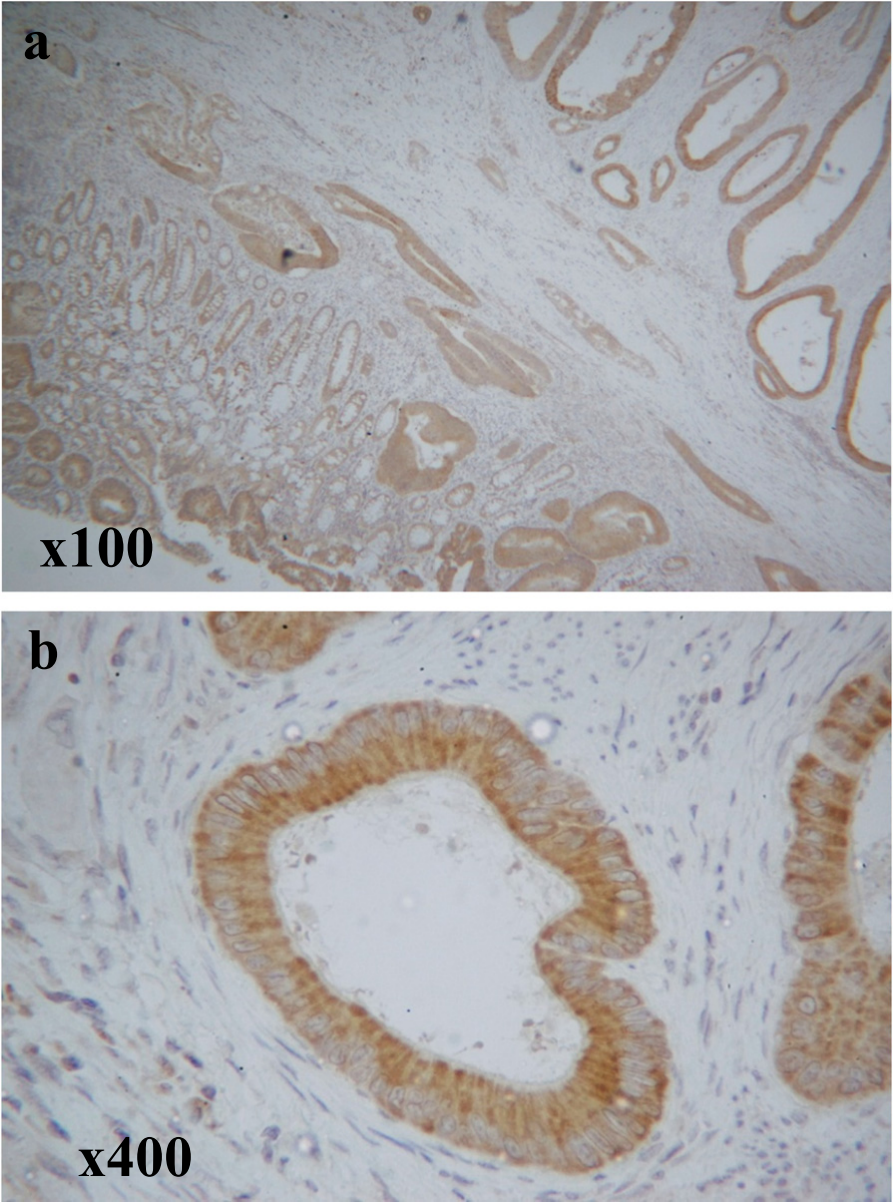
Immunohistochemical staining for PlexinB1. The immunohistochemical evaluation of PlexinB1 positive cells in colorectal cancer. The positive staining of the tumor gland was observed in the cytoplasm of cancer cells. **a** The photograph was taken at a magnification of  ×100, **b** The photograph was taken at a magnification of ×400

Positive PlexinB1 staining of the tumor gland was observed in the cytoplasm of cancer cells (Fig. 2). The staining intensity of epithelial tumor cells (in comparison to non-tumor cells) was scored as follows: 0 (no staining), 1 (weak staining), 2 (moderate staining) and 3 (strong staining). For the statistical analyses, scores of 0 or 1 were defined as negative, and a score of 2 or 3 was defined as positive [9].

### Statistical analysis

The associations between the expression of Sema4D and PlexinB1 and various clinicopathological factors were assessed using the χ2 test or Fisher’s exact test. To investigate the associations between relapse-free survival and various clinicopathological factors, a univariate survival analysis was performed using the Kaplan-Meier method, and the differences were evaluated using the log-rank test. A multivariate survival analysis was performed using Cox’s proportional-hazard model. Hazard ratios and 95 % confidence intervals were used to measure associations. The JMP® 10 software program (SAS Institute Inc., Cary, NC, USA) was used for all of the statistical analyses. *P* values of <0.05 were considered to be statistically significant.

## Results

### The correlations between Sema4D and PlexinB1 expression and the clinicopathological findings

The immunohistochemical staining of colorectal cancer tissue specimens revealed that 95 (42 %) and 105 (46.4 %) of the specimens were positive for Sema4D and Plexin B1, respectively. The expression of Sema4D was significantly related to stage, the depth of tumor invasion, lymph node metastasis, lymphatic invasion, and venous invasion (Table 1). Age, gender and histology did not significantly affect Sema4D expression. The expression of PlexinB1 was significantly related to stage, depth of tumor invasion, lymph node metastasis, lymphatic invasion, and venous invasion. There were no significant differences among the other factors.

**Table 1:** Correlations between clinicopathological findings and the expression of Sema4D and PlexinB1

Variables	Sema4D expression		*p* value	PlexinB1 expression		*p* value
	Positive	Negative		Positive	Negative	
	(*n* = 95)	(*n* = 131)		(*n* = 105)	(*n* = 121)	
Age (years)						
Mean ± SD	65.2 ± 12.3	67.2 ± 10.5	0.426	67.6 ± 11.0	65.8 ± 11.5	0.23
Gender						
Male	51(53.7)	73(55.7)	0.76	56(53.3)	68	0.666
Female	44(46.3)	58(44.3)		49(46.7)	53	
Stage						
I	15(15.8)	43(32.8)	0.001	16(15.2)	42	<0.001
II	21(22.1)	36(27.5)		19(18.1)	38	
III	59(62.1)	52(36.7)		70(66.7)	41	
Histology						
Well/mod	82(86.3)	120(91.6)	0.206	91	111	0.217
Other	13(13.7)	11(8.4)		14	10	
Depth of tumor invasion						
T1/T2	18(18.9)	50(38.2)	0.001	20	48	0.006
T3/T4	77(81.1)	81(61.8)		85	73	
Lymph node metastasis						
negative	37(38.9)	78(59.5)	0.001	35	79	<0.001
positive	58(61.1)	53(40.5)		69	42	
Lymphatic invasion						
Negative	18(18.9)	66(50.4)	<0.001	21	57	<0.001
Positive	77(81.1)	65(49.6)		84	64	
Venus invasion						
Negative	70(73.7)	117(89.3)	<0.001	76	111	<0.001
Positive	25(26.3)	14(10.7)		29	10	

*Sema4D* semaphorin 4D. *Other* poorly-differnetiated, mucinous-type, small-cell, signet-cell

### The correlations between Sema4D and PlexinB1 expression

Among 85 tumors which were found to be positive for Sema4D, 63(74.1 %) were positive for PlexinB1. On the other hand, among the 131 tumors which found to be negative for Sema4D, 42(32 %) were negative for PlexinB1. There was a significant correlation between the expression of Sema4D and PlexinB1 (*p* < 0.001, Table 2).

**Table 2:** Correlation between Sema4D and PlexinB1 expression in colorectal cancer

	Sema4D positive	Sema4D negative
	(*n* = 85)	(*n* = 131)
PlexinB1 positive	63(27.9)	42(18.6)
(*n* = 105)		
PlexinB1 negative	32(14.1)	89(39.4)
(*n* = 121)		

*Sema4D* semaphorin 4D

### The correlations between positivity for both Sema4D and PlexinB1 and the clinicopathological findings

Sixty-three patients (27.9 %) expressed both Sema4D and PlexinB1. There were no significant differences in the amount of infiltrated inflammatory cells around the tumor stroma between the positive Sema4D/PlexinB1 group and the negative Sema4D/PlexinB1 group (*p* = 0.539). We examined the correlations between the positive expression of both Sema4D and PlexinB1 and the clinicopathological findings. The expression of both Sema4D and PlexinB1 was found to be correlated with all of the clinicopathological factors that were examined, with the exception of age, gender and histology (Table 3). In addition, we evaluated the presence of any correlation between the treatment as an adjuvant chemotherapy and the expression of Sema4D and PlexinB1. Adjuvant chemotherapy was administered to patients who belonged to the high risk groups with stage II and stage III disease, except for patients who rejected any further treatment and those who demonstrated a poor performance status. As a result, no significant differences were observed between the adjuvant chemotherapy and the combination of Sema4D and PlexinB1 expression at each stage (Table 4).

**Table 3:** Correlations between clinicopathological findings and the combination of Sema4D and PlexinB1 expresion

Variables	Sema4D and PlexinB1 expression		*p* value
	Both positive	Others	
	(*n* = 63)	(*n* = 163)	
Age (years)			
Mean ± SD	68.1 ± 10.2	66.0 ± 11.7	0.351
Gender			
Male	35	89	0.897
Female	28	74	
Stage			
I	7	51	<0.001
II	11	46	
III	45	66	
Histology			
Well/mod	53	149	0.123
Other	10	14	
Depth of tumor invasion			
T1/T2	10	58	0.002
T3/T4	53	105	
Lymph node metastasis			
negative	18	96	<0.001
positive	45	67	
Lymphatic invasion			
Negative	9	69	<0.001
Positive	54	94	
Venus invasion			
Negative	41	146	<0.001
Positive	22	17	

*Sema4D* semaphorin 4D. *Other* poorly-differnetiated, mucinous-type, small-cell, signet-cell

**Table 4:** Correlations between adjuvant chemotherapy and the combination of Sema4D and PlexinB1 expression

Variables	Total	Sema4D and PlexinB1 expression		*p* value
	(*n* = 226)	Both positive	Others	
		(*n* = 63)	(*n* = 163)	
Stage I				
Adjuvant chemotherapy				
Treated	0(0)	0(0)	0(0)	-
Untreated	58(100)	7(100)	51(100)	
stage II				
Adjuvant chemotherapy				
Treated	13(22.8)	3(27.3)	10(21.7)	0.698
Untreated	44(77.2)	8(72.7)	36(78.3)	
Stage III				
Adjuvant chemotherapy				
Treated	81(73)	35(77.8)	46(69.7)	0.106
Untreated	30(27)	10(22.2)	20(30.3)	

*Sema4D* semaphorin 4D

### Measurement of the overall survival and relapse-free survival

**Fig. 3:**
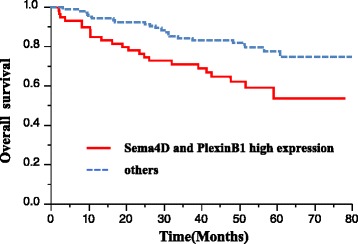
Kaplan-Meier estimates of over all survival among colorectal cancer patients with the positive expression of both Sema4D and PlexinB1

**Fig. 4:**
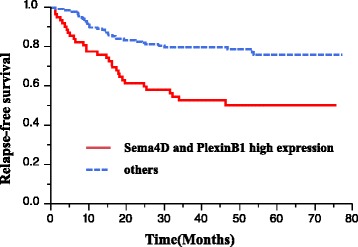
Kaplan-Meier estimates of relapse-free survival among colorectal cancer patients with the positive expression of both Sema4D and PlexinB1

The patients of both Sema4D and PlexinB1 positive groups exhibited a worse prognosis compared to the others (*p* < 0.001, Fig. 3). In the univariate analysis, histology, the depth of tumor invasion, lymph node metastasis, lymphatic invasion, venous invasion and positivity for both Sema4D and PlexinB1 were found to be significantly associated with overall survival (Table 5). However, a multivariate analysis demonstrated that the depth of tumor invasion (HR 2.692, CI 1.022–9.317; *P* = 0.044), lymph node metastasis (HR 2.304, CI 1.138–5.069; *P* = 0.019), and the positive expression of both Sema4D and PlexinB1 (HR 1.681, CI 1.004–2.819; *P* = 0.047) were an independent risk factors for worse survival (Table 5). The recurrence rate was 46 % (29/63) in patients who positively expressed both Sema4D and PlexinB1, and 16.6 % (27/163) in the other patients; this amounted to a significant difference (*p* < 0.001). The relapse-free survival (RFS) of the patients who positively expressed both Sema4D and PlexinB1was significantly worse than that of other patients (*p* < 0.001, Fig. 4). In the univariate analysis, histology, the depth of tumor invasion, lymph node metastasis, lymphatic invasion, venous invasion and positivity for both Sema4D and PlexinB1 were found to be significantly associated with RFS (Table 6). However, a multivariate analysis demonstrated that lymph node metastasis (HR 2.783, CI 1.425–5.822; *P* = 0.002), and the positive expression of both Sema4D and PlexinB1 (HR 1.079, CI 1.013–2.868; *P* = 0.044) were an independent risk factors for worse survival (Table 6).

**Table 5:** The results of univariate and multivariate analyses of the expression of Sema4D and PlexinB1, clinicopathological findings and overall survival in the patients with colorectal cancer

	Univariate analysis			Multivariate analysis		
Variables	Hazard ratio	95 % CI	*p* value	Hazard ratio	95 % CI	*p* value
Gender	0.928	0.574–1.509	0.762			
Male vs. Female						
Age	1.365	0.841–2.207	0.205			
≧70 vs. <70						
Histology	2.666	1.391–4.373	0.004	1.758	0.901–3.205	0.094
Other vs. well/mod						
Depth of tumor invasion	4.574	2.032–13.08	<0.001	2.692	1.022–9.317	0.044
T3/T4 vs.T1/T2						
Lymph node metastasis	4.469	2.432–9.017	<0.001	2.304	1.138–5.069	0.019
Positive vs. negative						
Lymphatic invasion	3.108	1.622–6.725	<0.001	1.318	0.603–3.235	0.505
Positive vs. negative						
Venus invasion	2.6	1.554–4.25	<0.001	1.409	0.813–2.394	0.216
Positive vs. negative						
Sema4D and PlexinB1 expression	2.567	1.587–4.158	<0.001	1.681	1.004–2.819	0.047
Both positive vs. others						

*Sema4D* semaphorin 4D. *Other* poorly-differnetiated, mucinous-type, small-cell, signet-cell. *CI* confidence interval

**Table 6:** The results of univariate and multivariate analyses of the expression of Sema4D and PlexinB1, clinicopathological findings and relapse-free survival in the patients with colorectal cancer

	Univariate analysis			Multivariate analysis		
Variables	Hazard ratio	95 % CI	*p* value	Hazard ratio	95 % CI	*p* value
Gender	0.966	0.598–1.617	0.966			
Male vs. Female						
Age	1.508	0.922–2.473	0.106			
≧70 vs. <70						
Histology	2.845	1.480–5.081	0.002	1.743	0.894–3.178	0.099
Other vs. well/mod						
Depth of tumor invasion	4.062	1.985–9.781	<0.001	2.12	0.911–5.824	0.083
T3/T4 vs.T1/T2						
Lymph node metastasis	4.871	2.729–9.358	<0.001	2.783	1.425–5.822	0.002
Positive vs. negative						
Lymphatic invasion	3.848	2.001–8.344	<0.001	1.658	0.740–4.129	0.227
Positive vs. negative						
Venus invasion	2.183	1.217–3.729	0.01	1.049	0.569–1.856	0.873
Positive vs. negative						
Sema4D and PlexinB1 expression	2.647	1.611–4.326	<0.001	1.079	1.013–2.868	0.044
Both positive vs. others						

*Sema4D* semaphorin 4D. *Other* poorly-differnetiated, mucinous-type, small-cell, signet-cell. *CI* confidence interval

## Discussion

The expression of Sema4D and PlexinB1 has been shown to be related to the prognosis of patients with various malignancies. Wang et al., reported the expression of Sema4D to be a novel indicator of a poor prognosis in CRC patients [8]. Kato et al., reported that the positive expression of both Sema4D and PlexinB1 was significantly correlated with worse survival in patients with pancreatic cancer [9].

On the other hand, it was reported that the decreased expression of Sema4D and PlexinB1 was associated with local recurrence and poor prognosis in breast cancer [10]. Although, the expression of Sema4D and PlexinB1 have been investigated in some solid malignancies, the correlation between the expression of these factors and the prognosis of patients with malignant tumors remains controversial.

Angiogenesis and invasive growth are induced through binding to Sema4D and PlexinB1. Consequently, the Sema4D and PlexinB1 positivity increases the possibility of relapse. Two pathways downstream to PlexinB1 have been reported to be mechanisms which underlie angiogenesis and invasive growth. The first mechanism is the transactivation of the tyrosine kinase activity of Met [12], a tyrosine kinase receptor which mediates invasive growth [13], the other is the activation of small GTPase Ras homolog gene family member A (RhoA) [14], Class IV semaphorins promote angiogenesis by stimulating Rho-initiated pathways through Plexin-B, and the phosphorylation of MAPK and Akt [15]. The interaction of these signal cascades contributes to the progression of cancer. As a result, the combination of the two mechanisms reflects tumor progression and a worse RFS.

Sema4D can bind to several receptors and induce various effects [16]. CD72, a member of the C-type lectin family, is a low-affinity Sema4D receptor that is expressed on immune cells, such as B cells, dendritic cells, macrophages and mast cells. The interaction between the immune cells promotes the aggregation and survival of B cells, and enhances the activation of B cells during antibody production [6]. The high-affinity Sema4D receptor plexinB1 is mainly expressed on endothelial cells and epithelial cells and promotes their motility [7, 17]. Plexin B2 receptors have a low-affinity for Sema4D, and are involved, with the mediation of dendritic epidermal T cells, in the wound healing process in the skin [18, 19]. Thus, Sema4D receptors are located in various places and have many roles. Above all, PlexinB1 receptors have a high affinity for Sema4D, and contribute to tumor progression. As a result, it is a factor that may cause recurrence.

Kato reported the cells expressing Sema4D in the tumor stroma of pancreatic cancer to be tumor-infiltrating lymphocytes(mainly T cells and B cells) [9]. In this study, we could not clearly elucidate the cell in which Sema4D is expressed, although, we consider that Sema4D is also expressed in the T cells and B cells in the tumor stroma of colon cancer. It has recently been hypothesized that Sema4D is involved in the regulation of the immune response in the tumor microenvironment. Evans et al. demonstrated that Sema4D creates a barrier to immune infiltration and affects the balance of regulatory and effector immune cells and signals. These immunomodulatory functions promote tumor progression [20]. Thus, there is a possibility that cancers in which Sema4D and PlexinB1 is expressed have enhanced invasive capacity through their control of the host immune response and that they may, as a consequence, cause a relapse.

In this study, the combined expression of Sema4D and PlexinB1 was found to be an independent risk factor for disease relapse in the multivariate analysis (Table 5). Although no significant differences were observed between the adjuvant chemotherapy and the combination of Sema4D and PlexinB1 expression at each stage, the relapse-free survival of the patients who positively expressed both Sema4D and PlexinB1 was significantly worse than that of other patients (Fig. 3). As a result, the positive expression of both Sema4D and PlexinB1 was thus considered to be closely associated with recurrence. Although the evaluation of the individual expression of either Sema4D or Plexin-B1 was an indicator of malignant potential [8, 21], it is thought that the combination of both Sema4D and PlexinB1 is a more accurate predictor of recurrence in CRC patients.

While Sema4D and PlexinB1 may represent sensitive biomarkers for helping to select patients who are at a high risk of early relapse, our retrospective data analysis didn’t support attempting to definitively link their expression to predicting patient outcomes as a function of postoperative adjuvant chemotherapy (Table 4). Therefore, further studies on the effects of Sema4D and PlexinB1 are needed for evaluating their relevance regarding selecting patients for postoperative adjuvant chemotherapy.

## Conclusion

Our results indicated that the detection of the combination of Sema4D and PlexinB1 was useful for predicting disease recurrence in CRC patients. However, further studies will be necessary to more definitively investigate whether the detection of the combination of Sema4D and PlexinB1 may be useful for selecting cases in which postoperative adjuvant chemotherapy will be efficacious after curative resection.

## Abbreviations

CRC, colorectal cancer; DAB, diaminobenzidine; MSI, microsatellite instability; MT1-MMP, membrane type 1-matrix metalloproteinase 1; PBS, phosphate-buffered saline; Sema4D, Semaphorin4D.
